# Abstracts and Gleanings

**Published:** 1882-01-20

**Authors:** 


### ABSTRACTS AND GLEANINGS.

### NOTES ON THE PREPARATION AND PROPERTIES OF SOME NEW REMEDIES.

**Paraffin Ointments and Oleates.**—All cerates and ointments, especially those subject to becoming soon rancid, keep well if made up with some soft paraffin base, such as is now sold under the name of petrolina, or with the paraffin ointment proposed in the printed report of the committee of the American Pharmaceutical Association on the Revision of the United States Pharmacopœia for 1880.

A subnitrate of bismuth ointment made up with such a base is considered as much better in many cases than the more commonly used zinc ointment.

A series of oleates will doubtless be officinal in the United States Pharmacopœia for 1880, the oleate of mercury having already been in use for some years. The oleates of the alkaloids, such as of aconitia two per cent., morphia five per cent., quinia twenty-five per cent. and veratria two per cent., furnish elegant methods of administration.

**New Poultice.**—A new form of poultice has been introduced by a French chemist as a substitute for linseed and other ordinary poultices. It consists of an extract from Irish moss (*Fucus crispus*) dried between sheets of cotton wool. For use, a piece or suitable size is cut and dipped in boiling water, until quite swollen; then applied to the part, and covered with the accompanying piece of gutta-percha sheeting. It possesses the great advantages of being cleanly, of not drying quickly, of not easily slipping from its place and of not having any unpleasant odor, as well as of being so quickly and simply prepared. It is offered in packages containing sheets five by eight inches and eight by thirty.

**Elastic Caustic Crayon.**—Elastic crayons of nitrate of silver can be prepared by dipping small laminaria tents in thick mucilage, and then rolling them in finely-powdered lunar caustic. When dried it makes crayon which can be introduced into a cavity without fear of breakage. Other caustics can be used in like manner.

**Castor-Oil.**—Castor-oil may be so palatable that a patient will not recognize it, if it is made into an emulsion containing castor-oil ℥i., tinct. cardamon comp. ʒiv., ol. gaultheriæ gtt. iv., pulv. acaciæ and pulv. sacchari alb. aa ʒij., aq. cinnamoni q. s. ad ℥iv.; misce secundum artem. German children are even said to quarrel over the confection of castor-oil made into a paste with either about three parts of coarsely granulated sugar or two parts of comp. licorice powder, and flavored with a little powdered cinnamon or grated lemon peel.

**Kooso.**—Kooso, one of the most certain of tænicides for tapeworm, is prepared, unimpaired in its strength and in a form which does not excite repugnance, by treating ℥ss. of fresh powdered kooso with ℥i. of hot castor-oil, and afterwards with ℥ij. of boiling water by displacement, expressing and by means of the yolk of an egg combining the two percolates into an emulsion; adding gtt. xl. of ether, and flavoring with some aromatic oil. This emulsion is to be taken at one dose early in the morning, after a previous fast of nearly a day. The worm is usually expelled dead after about six to eight hours.

**Cinchonia and Quinia.**—Cinchonia can be very acceptably administered in the form of a troche if accompanied by a little bicarbonate of soda, so as to make the mixture alkaline, and thus prevent its solution and taste in the mouth. Quinine finds a very good solvent in milk, which almost completely disguises its bitterness if taken in the proportion of f. ℥i. to the grain. This mode of administering quinine is of especial use with children. A solution of quinine in glycerine made gr. i. to the f. ʒi. can be given in a cupful of milk without the child knowing it.

**Iron Albuminate.**—An albuminate of iron has been for some years in use in Germany containing about five per cent. of ferric oxide. It is a perfectly transparent, light brown liquid, nearly tasteless, which will keep well in cool weather for several weeks. It can be obtained from its solutions by precipitation with common salt, and this when dried and powdered is again readily soluble in water.

**Perfumed Carbolic Acid.**—Perfumed carbolic acid is prepared from carbolic acid one part, oil of lemon three parts, alcohol of thirty-six degrees one-hundreth parts mixed. This mixture, which appears to be quite stable, has only the odor of lemon, is what has been known as "Lebon's perfumed carbolic acid," the formula for which has long been kept secret, but has now been made known in the Moniteur Scientifique of Paris. The antiseptic properties are in no way affected by the oil of lemon.

**New Disinfectant.**—A new disinfectant has been introduced in Australia composed of one part of rectified oil of turpentine and seven parts of benzine, with five drops of oil of verbena to each ounce of the mixture. Its purifying and disinfecting properties are due to the power possessed by its ingredients of generating peroxide of hydrogen or ozone Articles of clothing, furniture, wall-paper, books, and papers may be saturated with it without damage. When it has once been freely applied to any rough or porous surface its action persists for an almost indefinite period. This may be shown readily at any time by putting a few drops of a solution of iodide of potassium upon the surface which has been disinfected, when the ozone, which is being continually generated, will quickly liberate the iodine from the combination with the potassium, giving rise to a yellow discoloration, or a blue if boiled starch has been added to the iodide of potassium solution.

**Suberin.**—An impalpable cork powder under the name of suberin has come into use for the treatment of chapped nipples and other like purposes. It is dusted on after first washing the nipple, and then covered with a gold-beater's skin, cut star-shaped, in the centre of which punctures are made with a needle. When the child is suckled the powder is washed off with water, and the skin replaced, the child drawing the milk through that without giving pain. After each nursing the powder is dusted on again, and the gold-beater's skin placed over it. It is also being used for the chafing in children instead of lycopodium, being preferred on account of containing tannin, and also costing much less.

**Odorless Iodoform.**—The odor of iodoform is very much disguised by the presence of the volatile oils, such as peppermint and cloves, and also by balsam of Peru. Five to eight drops of the oil of fennel to the gram of iodoform is considered, however, to be the most effectual.

**Pyrogallic Acid.**—Ointment of pyrogallic acid is being used instead of that of chrysophanic acid in many cases with good results. Psoriasis is the disease for which it has been chiefly tried, and the most convenient strength has been found to be that of ten per cent. Most of those who have tried it—for instance, Prof. Hebra—prefer the remedy to chrysophanic acid. They find that, though its action is slower, it has the advantage over the latter in exciting scarcely any inflammation in the part to which it is applied, and in staining the skin but slightly, the brown color produced by it quickly disappearing. Hebra has never yet seen any poisonous symptoms follow its application to the skin, although in his cases it could always be detected in the urine. A case has, however, been lately reported by Dr. A. Neisser, where a patient died with the symptoms of pyrogallic acid poisoning in the skin clinic at Breslae. One half of the body of a robust man having been eovered with a chrysophanic acid, and the other half with a ten per cent. pyrogallic acid ointment, he was attacked with vomiting, and died in collapse on the fourth day. The urine was dark brown, and had a thick sediment, which consisted of a very abundant blackish-brown substance, partly amorphous, and partly in the form of casts, but containing no blood cells. As the spectrum showed the characteristic bands of hæmoglobin, and similar debris to that in the urine was found in the blood itself and in the renal tubules, there could be no doubt that it consisted of disintegrated red blood corpuscles. Dr. Neisser explains its poisonous action by its activity for oxygen in the presence of alkalies, and the consequent destruction of the red blood corpuscles, which are the carriers of oxygen. He considers that its use had best be restricted to the head and face, while chrysophanic acid may be used upon the surfaces covered by the clothing.—[Boston Medical Journal

---

**Was the Thoracic Duct Injured in the Case of President Garfield ?**—It is a curious feature in some of the criticisms and comments on the President's case, that, in view of the ante-mortem symptoms and the post-mortem examination, it should have been asserted that the "rapid emaciation and mal-nutrition" were due to a wound of the thoracic duct. As distinguished a physician as Dr. Wm. Hunt says (Medical News and Abstract, November, 1881): "There was the origin of the thoracic duct, with its receptaculum chyli, right in the line of the wound; hence, the rapid emaciation, and the other nutritive disturbances, further explained by disturbance of the sympathetic trunks and ganglia."

The official record of the post-mortem examination (American Journal of the Medical Sciences, October, 1881), shows, by statement and diagram, the track of the ball in the spinal column to have been by a small aperture of entrance, one-quarter of an inch distant from the intervertebral foramen of the twelfth dorsal and first lumbar vertebra, and the centre of the foramen of exit to lie one-half of an inch to the left of the median line. With a view to a determination of the exact relations of the parts, I have made, within the past week, two dissections at the University of Pennsylvania, studying with especial care the thoracic duct and receptaculum chyli, and the structures immediately adjacent.

The following is a summary of my observations: The receptaculum lay in one case directly upon the median line; in the other, about a line to the right of the median line, closely applied to the body of the second lumbar vertebra, commencing in a pouch at its inferior border, and tapering upwards and to the right, to terminate at the thoracic duct at the lower border of the first lumbar vertebra. The thoracic duct then applied itself closely to the right crus of the diaphragm, and passed up through the aortic opening. The whole of this structure was efficiently protected, anteriorly and to the left by the abdominal aorta, and to the right by the vena cava.

Spicules of bone could not then have reached it from either side, where it was protected by more important organs; and to have been injured posteriorly requires the supposition of more extensive injury to the vertebra than has been stated.

The following summary of symptoms present in the six recorded cases of wound of the thoracic duct, is given by Bradley ("Injuries and Diseases of the Lymphatic System," London, 1879) and is of interest in connection with the study of this historic case.

"Hoffmann's first case was that of a woman wounded through the left side with a knife. Following the wound there was a copious discharge of a spontaneously coagulating fluid, which was observed to be milky during digestion, and clear while the patient was fasting. In his second case, the eseape of chyle followed the opening of an abscess of the posterior mediastinum. Monro relates a case where the thoracic duct was wounded by a stab; the lymph escaped externally and also into the pleural cavity, interfering with the heart's action. Guiffort's case is of a similar nature.

Bonnet gives the history of a Baron Heinden, who was wounded in battle by a bullet, which escaped beneath the left scapula. From this wound there gradually began to flow an excessive quantity of lymph, 'tanta in copia effluxises, ut non solum lintea quintuplicata, indusium lodicesque imbuerit, sed quoque limbos inundaverit.' The patient lived for several months, dying at last of exhaustion."

In Quinck's case, "the pleural cavity became so full of extravasated lymph that paracentesis had to be performed to prevent suffocation, from which, indeed, the patient eventually died."

It will be seen from the above, that a stillicidum of lymph, generally in great quantities, is the invariable attendant of wounds of this nature. The fact that this symptom was wanting, so far as we know, in the President's case, precludes, with even greater certainty than the anatomical considerations, the possibility of any injury to this most important organ.—[HOWARD ATWOOD KELLY, M. D., in Medical News.

**Cold Water Enemeta in Diarrhœas of Children.**—In the American Journal of the Medical Sciences, No. 151, for July, 1878, (page 133), Dr. Michael J. B. Messemer, gives an article entitled "Cold Water Enemata as a Therapeutic Agent in Chronic Diarrhœa," in which he cites a number of cases, showing its remarkable efficacy. I was immediately impressed with the idea that said treatment deserved a thorough trial: one of the reasons being the fact that our therapeutical knowledge of astringents, etc., in these cases, was not sufficient, for, do what I could, some cases remained in *statu quo* or died. Children are by far the greater sufferers, and when called to one I felt frequently as if I wished some brother who was pining for a case had the call. I had been in the habit of using enemas of starch and opium, and since it had been of benefit, the idea that a vehicle lighter than starch, which at the same time cooled the surface of the inflamed mucous membrane of the bowel, would be very good, came to me, and I heartily thanked Dr. Messemer for extending a helping hand to one very frequently in the "slough of despond." Now, I have since first reading that article, used enemas of cold water, and cold water with tinct. opium, and the results have been such that I feel it my duty to send in this article; it having failed only in cases where my patient was beyond recovery, or when attendants failed to use as directed. I will cite a few cases:

CASE 1.—J. S., male, aged two and a half years; had had diarrhœa for several months, and I was called September 1st, 1879. I found the child suffering from chronic diarrhœa, with all the attendant symptoms. I advised an enema of cold water after each action, first injecting then pressing on the abdomen gently until the water was passed; then injecting again. This, with a strict regulation of diet, and a course of tonics, he being much debilitated, formed the treatment. This course, followed with remarkable perseverence, cured the child in a few weeks. Case had been treated with everything in materia medica generally found useful in such cases, but was gradually giving away, when above plan was used.

CASE 2.—Called December 31st, 1879, to G., male, aged three years. Found a case of acute dysentery. His father had exhausted every domestic remedy, having used both castor oil and salts, with tinct. opium, and a host of teas informant knows not of; still the child had dysenteric discharges with tormina, tenesmus, etc. I advised the use of cold water and tinct. opium (laudanum

ʒj. to Oss water), as an enema, telling him to use the syringe after each dejection, immediately. I also left a prescription of sub. nit. bismuth, etc., to be used if the injections failed. But the dysentery yielded at once, and nothing was given per orem.

CASE 3.—Called to a family where two ladies and two children were suffering with dysentery, cases running from eight to ten days to within two days of the time I was summoned. Dr. M., physician in charge, had exhausted every remedy he possessed any knowledge of, and still the trouble continued. I advised the cold water and laudanum. as above, in all four cases, and I did not visit either of the cases a second time, for they began at once to get better, and were soon convalescent.

Now, with Dr. Messemer, I am not in favor of discarding everything else, and trusting cold water alone; but I beg physicians everywhere to try the above, assuring them that they will benefit many cases otherwise incurable. Read Dr. Messemer's article; it will repay all trouble and expense.

Of course, in treating either of the above troubles it is absolutely necessary to clear the bowel of accumulations of fæces, etc.

The greatest trouble will be in getting mothers to diet the patient properly. With these remarks, and a repeated request to try cold water, I close an article already too long.—[Medical and Surgical Reporter.

[We presume the writer does not mean to say in the above that the entire quantity of the water (Oss.) containing ʒj. of laudanum was injected in a child three years old, but he should be more explicit.—ED. RECORD.]

**Explosive Mixtures.**—Medical men but rarely pretend to be good chemists, for it would require longer devotion to chemistry than the average medical student can afford; thus can it be marveled at when we see formulas and prescriptions that, if dispensed according to the wishes of the prescriber, would result in an incompatible combination and often explosive compounds! Thinking it not ill placed to perhaps refresh the memory of the profession regarding such mixtures, especially explosive mixtures, we have selected some examples and formulas that when combined in certain proportions become dangerous and in many instances have ended seriously; they are examples that have been experimented with, some intentionally, while others were prescribed by a badly informed physician and dispensed by a very incompetent druggist.

1 Chlorate of potash, powdered galls, tannic acid. M. Ft. pulvis.—To be used for a gargle. The powders should be mixed separately with water and not rubbed all together.

2. Chlorate of potash and pulv. catechu.—This combination is intended as a dentifrice. It however should not be dispensed alone. If other combinations are made, the danger is averted.

3. Chlorate of potash, hypophosphite of soda and water.—If the salts are rubbed together, they will explode, but if dissolved separately in the water and mixed, no harm results.

4. Chlorate of potash, tannic acid, glycerin and water.

If the tannin, chlorate of potash and glycerin are rubbed together an explosion ensues, but if the acid is first dissolved in the glycerin and the chlorate of potash in the water and mixed, no harm follows.

5. Chlorate of potash, Tr. ferri chlor. and glycerin, half an ounce of each.

This combination, so often used, when put together in the above proportions, is very liable to explode, especially if warmed.

6. Soda chlor. 2 dr.: antimon. sulph. aurat. 20 gr.

This combination, if even gently triturated, is liable to inflame with a crackling noise.

7. Lac. sulphuris 3 gr., antimon. sulph. aurat. ½ gr., zinci valer. 2 gr., potass. chlor. 2 gr. M. Ft. Pulvis, Make 10 alike.

Potash permanganate, when associated with any readily oxidizable substance sueh as glycerin, explodes.

8. Chromic acid 10 gr., glycerin 1 dr.

This mixture is liable to explode, unless the glycerin is added to the acid drop by drop.

Iodine and ammonia form a very powerful explosive agent when combined, unless some water is introduced into the mixture, which seems to retard the development of nitrogen iodide, upon which the explosive properties depend.

9. Iodine ʒss., linim, camph. co., linim. saponis aa℥ii. M. F. Linim.

This combination exploded once in the hands of a pharmacist from the iodine and the ammonia in the liniment camph. co. coming in contact.

10. Acidi nitrici; acidi muriatici; Tr. nucis vom., aa ʒij. M.

This prescription was once ordered by a physician, and exploded after several hours.

11. Acid. nitro-mur., ℥j, Tr. cardamomi ℥ss M.

Also this combination was the result once of a serious injury. —[Pacific Medical Journal.

**Is There a Specific Urethritis ?**—In a "special article" in the September number of the New York Medical Journal and Obstetrical Review, Dr. P. Albert Morrow handles the question of the specific or non-specific nature of gonorrhœa. After a fair statement of close analysis of the arguments for and against specificity, he concludes that the position of the virulists rests altogether upon pure hypothesis, and is wholly untenable, while all the facts—experimental, clinical and pathological—are overwhelmingly in favor of the non-specific character of gonorrhœal inflammation. When we apply the gauge of specificity to gonorrhœa it corresponds to none of the conditions of an undoubtedly specific inflammation. No artificial production of any disease belonging to this group is possible; a specific disease is the product alone of a specific poison. Gonorrhœa, on the contrary, may be due to a variety of causes—contagious, irritant (mechanical or chemical), diathetic, etc. Again, in all specific diseases there is between the time of infection and the first expression of the disease a period of incubation. No incubation, properly so called, characterizes gonorrhœa. A drop of this same gonorrhœal pus, which may require two or three days to excite suppuration of the urethra, will develop such effect in a few hours when applied to the conjunctiva, showing that the so-called incubation depends not upon the quality of the exciting cause, but upon the susceptibility of the mucous membrane. Another distinctive peculiarity of this group is that a single attack of the disease confers almost complete security from another attack—a peculiarity precisely the opposite of what is observed of gonorrhœa. The morbid poison of a specific inflammation, once in action, continues until the textual predisposition to its special stimulus is exhausted. The patient is incapable of regenerating the poison or of being affected by it when exposed anew. Both of these conditions are negatived in the clinical history of gonorrhœa. Finally, specific inflammation determines special pathological changes and demands special treatment. Identical pathological processes are met with in urethritis from causes, and the most radical of virulists treat all urethral inflammations alike.—[Mississippi Valley Medical Monthly.

**Select Formulæ for Ingrowing Toe-Nails.**—Cut a notch about the shape of a V in the end of the nail, about one-quarter of the width of the nail distant from the ingrowing side. Cut down as nearly to the quick as possible, and one-third the length of the nail. The pressure of the boot or shoe will tend to close the opening you have made in the nail, and thus affords relief. Allow the ingrowing portion of the nail to grow without cutting it until it gets beyond the flesh.

To keep the flesh from the nail will effect a cure. A strip of lint covered with simple cerate may be carefully inserted between the nail and flesh. But prompt recovery will most generally attend the use of collodion, painted over the sore part with a camel's hair brush.

Make a bridge of muslin from the great toe to the third, and allow the middle toe to rest on the bridge; this effectually removes the pressure from the great toe, and the parts eventually get well.

Do not remove any portion of the nail; let the patient wear a roomy shoe; once or twice a week scrape the entire surface of the nail lightly, pack a bit of cork or pledget of lint, by means of a fine knife-blade, under the corner of the nail, and let the entire nail, corners included, grow out clear of the toe, when it may be trimmed by cutting it off square. With a little patience and perseverance a radical cure may be expected.

After trimming the nail so as not to wear the stocking, scrape a narrow strip on the top of the nail from the skin to the front edge, as thin as possible; then cut out a V shaped piece in the centre of the edge, with the point of the V running in the thin scraped place just far enough not to draw blood. This, repeated once a week, has proved a permanent cure in every case tried, and has the advantage of being done by the patient at home.—[St. Louis Eclectic Med. Jour.

**Supra-Pubic Operation for Lithotomy.**—Dr. Petersen (Archiv. fur Klin. Chir., Band XXN. S 752) considers that the dangers of the high operation for stone, which consist in injury of the peritoneum and infiltration of urine, may be prevented by modern methods of operation. He has found, by observation on eleven bodies, that when Braune's method is followed by the gradual distention of the rectum, the full bladder is dragged further forward and upright, and the peritoneum thus rises considerably with its anterior fold, much more so than when this is not the case. Petersen, therefore, has recently always operated in such a manner that he not only fills the bladder to the utmost, but the rectum also, by the introduction of a rectal tube, and the gradual injection of water the same heat as the body, so as to dilate it. In his last two operations, Petersen did not even see the peritoneum; while in his earlier operations he was obliged to push it upward. He considers that the danger of infiltration of urine may be overcome by careful suture of the bladder with fine catgut, under complete antiseptic precautions.

The special indications for the high operation he sets out as follows: 1, The presence of a large hard stone. 2, Encapsuled stone. 3, Stone in diverticula, behind the prostate gland. 4, Enlargement of the prostate gland. 5, Hæmorrhoids. 6, Fat subjects. 7, Tumors of the bladder. 8, Impermeable stricture (with the assistance of posterior catheterization.)—[London Medical Record.

**Temperature of Sleeping-Rooms.**—Dr. Horace Dobell, of London, in his excellent work, "Winter Cough," makes remarks on the temperature of bed-rooms, that are so appropriate that I will quote them. He says: "But before leaving the subject of sudden changes of temperature, I must not forget to speak of sleeping-rooms. It is quite astonishing what follies are committed with regard to the temperature of sleeping-rooms. On what possible ground people justify the sudden transition from the hot sitting-room to a wretched cold bed-room, which may not have had a fire in it for weeks or months, it is impossible to say; but it is quite certain that the absurd neglect of properly warming bedrooms is a fruitful source of all forms of catarrh. We cannot too much impress this upon our patients." For those who do not become warm quickly after they go to bed, during cool or damp weather, the bed-clothes should be warmed by a hot smoothing-iron, or a warming bed-pan, before they retire for the night. This warming operation may be necessary even if there has been a fire in the sleeping-room all day. If a patient is subject to profuse night sweats the dampened bed-clothes should on each morning be removed from the bed, and fresh, well dried cotton clothes (linen sheets and pillow cases should be eschewed) supplied in their stead. If the perspiration has been slight, the bed sheets alone may be all that require removal, or even those may be so slightly dampened that their being placed before a grate fire will be sufficient to dry them for the next night's use.—Dr. Rumbold's Hygiene of Catarrh.—[Independent Practitioner.

**The Bacillus Lepræ.**—The recent descriptions of Neisser, Eklund and others, of the bacillus lepræ, first observed by Hansen, have awakened widespread interest in the pathology of leprosy. Dr. I. Bermann, of this city (Baltimore), has been able to detect the presence of the bacillus in leprous tissue, and to confirm the discoveries of the above-named authors. Sections exhibiting the bacillus were shown at the recent meeting of the American Dermatological Association at Newport. Since then, Dr. Bermann has improved his methods of preparation and investigation, and is now able to demonstrate the bacillus to perfection. His results were shown at a recent meeting of the Baltimore Clinical Society. The directions given by Neisser for detecting the little organisms enable one to recognize them, but not with satisfaction. Weigert's methods for investigating bacteria answer the purpose perfectly. The affinity of the bacillus lepræ for the aniline violet staining fluid is remarkable. By immersing the stained sections in oil of cloves for forty-eight hours, the violet color is removed almost completely from the tissues, leaving the bacilli stained dark purple and presenting sharp outlines. It remains very difficult to see them, however, even after this preparation, and objectives of high power (with small angle) must be employed. By using, as a condenser, an objective of about one-quarter inch, for illumination, they come out with splendid definition. The bacilli are mostly to be found in the protoplasm of the cells, but may be seen elsewhere. Sections thus prepared and examined will satisfy the most incredulous. Dr. Bermann has been the first in this country, we believe, to detect the bacillus, and will shortly publish his observations in full.—[Med. News.

**What Constitutes a Good Physician.**—A writer in The Spectator, says: "A man can hardly be a good physician without having a considerable share of native scientific ability; and he should also have much of the artist's sensibility to form, color, and expressive motion. The physician, besides actual accomplishments, of which he is likely to have quite enough outside his vocation, is often the most agreeable and well-informed man in the world, in his very large circle. And everything conspires at present to compel or invite the widening of his horizon. To be a really good physician a man must be a psychologist. This does not mean that if he finds in a case of insomnia no obvious cause for the patient's disorder, he should ask the unfortunate man if he has anything on his conscience, embezzlement, for instance. It is well known that Lord Eldon, when plain John Scott, and the lady whom he married, were lovers all their lives. Early in the honeymoon Mrs. Scott fell ill in a strange village, and the local practitioner was sent for. Having, after the usual routine, failed to make out what was the matter with this beautiful lady, he said: "'I am afraid, ma'am, there is something on your mind. You are not happy with your husband!" This was bad practice. There is but little necessity to allude to the dismissal of that doctor. The anecdote only illustrates, in passing, the sense in which the physician must not be a psychologist."—[Mich. Med. News.

**Black Sheep in the Flock.**—Very few suits for malpractice have occurred in San Francisco or in the state. Such suits depend commonly on the loose tongue and low appreciation of professional honor possessed by some cackling doctor. As a rule, this doctor is the cat in the meal tub. We have lately heard of a suit for malpractice in San Francisco which was so begotten. An ununited fracture was exhibited to a young surgeon. With that superior knowledge which every young surgeon—and not a few old ones—possess beyond every other surgeon, our juvenile Ambrose pronounced it a case of malpractice and suggested a lawyer. And a lawyer it was, and lawyer it is. So the profession stands before the public at the bar of the court, divided against itself and lowering its status in society. When will the professional instigators of suits for malpractice learn that they are blindly smiting themselves?—that they are undermining the house they live in?—that they cannot throw dirt on a professional brother without soiling their own hands? A little more professional honor, comrades! A little more professional sympathy and fraternity! A little more esprit du corps!—[Pacific Medical Journal.

[The same may be said of most of the professional criticisms in the case of President Garfield.—ED. RECORD.]

**Treatment of Spermatorrhœa.**—Dr. Nowatschek reports in Schmidt's Jahrbucker, January 1881, a case of spermatorrhœa consequent on typhoid fever, the diagnosis resting on the presence of spermatozoa in the fluid which was constantly oozing from the urethra. Iron, quinia and cold applications to the genitals were tried in succession with some success, but a cure was not accomplished. Lupulin, camphor, and bromide of potassium were without effect. Atropia was then employed, and the patient was completely cured in five days. The author cites a second case where he was equally successful with the hypodermic injection in the perineum of a one per cent. solution of atropia.—[Jour. de Med. de Paris, Oct. 8, 1881.

**What is Malaria?**—Touching the statement often made that there is no such thing as malaria, Dr. A. G. Smithe, of Baldwyn, Miss., says: "If malaria is a delusion I am ready to exchange the doctrine now held, whenever a better cause, (for a cause there must be,)for the diseases now charged to that cause is given. Call it what you may, poison, or a want of poison, give us something in exchange for our delusion."

That malaria—so-called—is a micro-organism breathed into the circulation through the lungs, may be now regarded as *almost* an established fact.—Ed. Record.

**New Hypodermic Syringe.**—Dr. Ely, in New York Medical Association, exhibited a hypodermic syringe revised and modified by himself, and manufactured by Tiemann, of New York. The instrument is commendable because of its compactness. The needle is inclosed in the piston, and the barrel has a close-fitting cap, which prevents the possible entrance of air or dust. The syringe is about as large as a lead-pencil.

**Bromide Ammonia in Cholera.**—Dr. E. Halsey Wood, in the Michigan Medical News, May 10, after sketching the phenomena of cholera, says of the Bromide of Ammonia: "It energizes the ganglia and restores innervation, and all the evidences of deranged function disappear under its influence. It is as specific in the mild as it is in the severe degree of gangliasihenia, and thus not only exhibits its potency but proves that the shape of disease assumed is due to different degrees of the same condition." In proof of his assertions the doctor describes a case each of diarrhœa, cholera morbus, cholera infantum, and Asiatic cholera, in which this remedy was successfully employed, and concludes with the assurance that it "is absolutely certain in its action. The administration of a dose imparts the sweet consciousness that it can be relied on to perform the assigned duty without failure in all cases. —[New York Medical Times.

**Collyrium for Dissolving Metallic Foreign Bodies from the Cornea.**—Dr. Rodriguez reports the following case, (Revista de Sciencias Medicas, Oct. 25, 1881): A blacksmith, aged eighteen years, while forging a piece of iron, received in his left eye a small splinter of the metal, which remained there incrusted in spite of all attempts to remove it. The following wash was then employed: Rose water, 90 grm.; Iodine, .05 grm.; Iodide of Potass., .05 grm.; the result was extremely satisfactory. The particle of metal was transformed into a soluble iodide of iron, and all traces of the foreign body had disappeared. The cornea regained its normal condition, and vision remained unaffected.—[Jour. de Med. de Paris.

**Dyspepsia Caused by Tight Lacing.**—Dyce Duckworth, M. D., in The Practitioner, says: "I find many cases of dyspepsia in women yield quickly to the use of proper stays. Again and again I have known chronic vomiting in young girls to be due solely to tight stays. Palpitation and dyspnœa, not due to anæmia, are frequently caused by bad stays. The worst cases naturally occur in young women who are inclined to embonpoint, and whether this be constitutional or aggravated, as is that condition by anæmia, the obese tendency commonly both adds to the compression and gives cause to the wearer to increase her troubles in the efforts to retain (what she conceives to be) shapely proportions.

**Improvement in Hypodermic Injection.**—Dr. Mason recommends the following as the best way of dealing with the piston of the hypodermic syringe when its packing gets worn and loose so that it does not work readily. Remove the small nut at the end of the piston and take half of the packing off (it is usually in two parts) and place between them a piece of chamois skin. Cut it round leaving it somewhat larger than the packing. It will absorb water, swell, and completely fill the barrel. A trial of this will convince the most skeptical of its value over all other devices to do away with the most annoying feature connected with the use of the syringe.—[Medical Times and Gazette.

